# Characterization of Holstein and Normande whole milk miRNomes highlights breed specificities

**DOI:** 10.1038/s41598-019-56690-7

**Published:** 2019-12-30

**Authors:** S. Le Guillou, A. Leduc, J. Laubier, S. Barbey, M.-N. Rossignol, R. Lefebvre, S. Marthey, D. Laloë, F. Le Provost

**Affiliations:** 1 GABI, INRA, AgroParisTech, Université Paris-Saclay, 78350 Jouy-en-Josas, France; 2 INRA, UE0326, Domaine expérimental du Pin-au-Haras, Exmes, France

**Keywords:** Next-generation sequencing, Gene expression, Epigenomics

## Abstract

The concept of milk as a healthy food has opened the way for studies on milk components, from nutrients to microRNAs, molecules with broad regulatory properties present in large quantities in milk. Characterization of these components has been performed in several species, such as humans and bovine, depending on the stages of lactation. Here, we have studied the variation in milk microRNA composition according to genetic background. Using high throughput sequencing, we have characterized and compared the milk miRNomes of Holstein and Normande cattle, dairy breeds with distinct milk production features, in order to highlight microRNAs that are essential for regulation of the lactation process. In Holstein and Normande milk, 2,038 and 2,030 microRNAs were identified, respectively, with 1,771 common microRNAs, of which 1,049 were annotated and 722 were predicted. The comparison of the milk miRNomes of two breeds allowed to highlight 182 microRNAs displaying significant differences in the abundance. They are involved in the regulation of lipid metabolism and mammary morphogenesis and development, which affects lactation. Our results provide new insights into the regulation of molecular mechanisms involved in milk production.

## Introduction

Milk is a complex secretory product and the source of nutrients for neonates and adults, whose composition could influence the short- and long-term health of consumers^1,2^. In addition to nutritional agents, it contains a large number of biological components, including microRNAs, small molecules involved in the post-transcriptional regulation of gene expression. Their roles in milk on recipient young animals are not well documented; many microRNAs, including those that are immune-related, are present and enriched in milk exosomes, membrane vesicles that deliver their content into recipient cells^3–5^.

Amongst fluids, milk presents the larger concentration of RNA and a higher variety of microRNA species^6^. Milk microRNAs are stable under adverse conditions, including RNase digestion, low pH, high temperature, and freeze/thaw cycles^3,7,8^. The milk miRNomes, studied in several species, including bovines, humans, marsupial tammar wallabies and giant pandas^9–12^, are highly conserved between species; however, some species specificities have been observed. Milk miRNomes vary considerably between individuals, depending on the maternal background (e.g., age and lifestyle) and health^13–15^. They also depend on the lactation period^10,11,16–19^ and even on different times of the day^20^.

The miRNomes of different milk fractions, i.e. fat, whey and cells, have been examined independently and compared within breeds^9^ or between species^21–23^. But the variation of the miRNomes of the whole milk, containing all milk fractions, according to the genetic background has still not been described. Moreover, concerning breed comparisons, three studies have characterized miRNomes in the mammary tissue of dairy and beef breed heifers^24,25^, on the one hand, and of two divergent phenotype swine breeds^26^, on the other hand. In all cases, the authors highlighted significantly differentially expressed microRNAs in the mammary tissues of the breeds.

If milk miRNome studies give information on microRNA which could act on recipient young animals, it is also a mirror of the mammary gland miRNome. In fact, most microRNAs present in milk originate from the mammary epithelium^11,27^, and a modification of mammary gland microRNA expression modulates its profile in milk^28^. In this context, a milk miRNome comparison of cattle breeds with different milk production characteristics will highlight microRNA essential for the regulation of genes involved in milk synthesis and secretion.

Holstein and Normande cattle are two dairy breeds with distinct milk production characteristics. The Holstein is the first world’s dairy breed, with the stronger production in terms of quantity. The Normande cattle is a hardy French breed, selected for the production of fat- and protein-rich milk, sought after for high-quality butter, cream and cheese manufacturing, and for their attractive meat properties. The Normande breed has lower milk production than the Holstein, but higher fat and protein content and higher adaptability to hardy conditions. Therefore, these two breeds are considered relevant models to investigate the differences in profiles of milk microRNAs in relation to milk traits. To date, the whole milk microRNA patterns of Holstein and Normande cattle remain unknown. In the current study, a high-throughput sequencing screen was conducted to characterize and compare the milk miRNomes of the two breeds, while also taking into account the impact of age. We aim to highlight microRNA regulators of molecular mechanisms involved in milk production.

## Results

### Dairy and milk quality traits

The Holstein and Normande cows had significantly different (p < 0.05) dairy milk production averaged over five weeks between breeds and for each age group, with higher production levels in Holsteins (Table 1). The Holsteins’ average milk production ranged from 22.3 kg/day for two-year-olds to 24.1 kg/day for three-year-olds. The Normandes’ average milk production ranged from 14.3 kg/day for two-year-olds to 21.2 kg/day for three-year-olds. Moreover, at two years of age, milk protein content is higher in Normandes than in Holsteins, e.g., 35.4 g/kg compared to 30.6 g/kg, respectively, but it does not differ between breeds at three years of age. While Normande milk is fat-rich, the fat content does not differ significantly between breeds, varying from 33 g/kg in the milk of three-year-old Holsteins to 43 g/kg in the milk of two-year-old Normandes. No difference in lactose, urea, acetone and BHB content was observed between breeds, whatever animal’s age. The somatic cell counts vary from 45,200 cells/ml to 150,600 cells/ml, with no significant differences between breeds (p < 0.05).

**Table 1:** Dairy and milk quality traits of experimental animals.

Age	Holstein	Normande	Holstein	Normande
	2 years (n = 4)	2 years (n = 3)	3 years (n = 5)	3 years (n = 5)
Dairy milk production averaged over 5 weeks (kg/day)	22.3 ± 2.4^a^	14.3 ± 2.2^b^	24.1 ± 1.4^a^	21.2 ± 1.8^b^
Protein content (g/kg)	30.6 ± 2.1^a^	35.4 ± 1.2*^,b^	32.5 ± 1.6^ns^	32.3 ± 2.7^ns^
Fat content (g/kg)	38.6 ± 5.0^ns^	43.4 ± 4.2*^,ns^	33.3 ± 4.6^ns^	37.4 ± 6.8^ns^
Lactose (g/kg)	482.5 ± 16.0^ns^	504.5 ± 26.2*^,ns^	503.8 ± 16.8^ns^	505.2 ± 21.4^ns^
Urea (mg/cl)	152.5 ± 53.2^ns^	95.0 ± 35.4*^,ns^	150.0 ± 35.4^ns^	130.0 ± 40.6^ns^
Acetone (mmol/l)	12.0 ± 5.4^ns^	12.0 ± 0.0*^,ns^	10.2 ± 1.8^ns^	9.2 ± 2.3^ns^
BHB (mmol/l)	8.8 ± 5.6^ns^	7.0 ± 0.0*^,ns^	5.8 ± 1.9^ns^	8.6 ± 4.4^ns^
Somatic cell count (cells/ml)	113,500 ± 139,189^ns^	126,000 ± 94,752*^,ns^	45,200 ± 26,119^ns^	150,600 ± 89,419^ns^

Values are expressed as the means with their standard errors. a, b: indicate a significant difference among breeds of the same age-group (p < 0.05, Mann & Whitney); ns: non-significant. n: number of individuals analysed; *: only two individuals analysed.

### Characterization of the Holstein whole milk miRNome

An average of 32,653,400 raw reads was obtained for the nine Holstein libraries, ranging from 4,641,274 to 56,924,058 raw reads per cow (Table 2). After library adaptors removal and size filtering (17–28 nt), 13.6 million clean reads on average were obtained, from 1,277,332 to 29,280,218 clean reads. They were aligned against the bovine genome (BosTau8), and the final mapped reads resulted in 9,299,953 reads on average, from 492,668 to 21,654,866.

**Table 2:** Sequencing data of whole milk microRNAs.

Holstein					Normande				
Individual	Age (years)	Raw reads	Cleaned^a^ and filtered^b^ reads	Mapped reads^c^	Individual	Age (years)	Raw reads	Cleaned^a^ and filtered^b^ reads	Mapped reads^c^
H1	2	23,959,434	11,207,607	7,442,391	N1	2	71,359,415	27,318,153	13,501,112
H2	2	29,605,886	12,894,672	8,424,745	N2	2	26,443,941	11,844,909	6,984,748
H3	2	4,641,274	1,277,332	492,668	N3	2	3,065,890	1,556,425	915,487
H4	2	10,057,132	5,613,708	3,677,686	N4	3	22,404,553	10,749,730	7,309,510
H5	3	56,924,058	29,280,218	21,654,866	N5	3	52,674,058	28,136,721	19,656,622
H6	3	43,242,357	13,344,848	9,917,574	N6	3	26,535,350	14,275,665	9,752,828
H7	3	33,041,730	15,520,982	10,350,694	N7	3	24,794,919	10,755,221	7,022,284
H8	3	56,840,975	23,475,553	15,295,377	N8	3	43,204,321	21,047,336	13,545,680
H9	3	35,567,753	9,386,785	6,443,574					
Mean		32,653,400	13,555,745	9,299,953	Mean		33,810,306	15,710,520	9,836,034

Average reads for each library on Holstein and Normande milk. a: library adapters removed; b: 17–28 nt size filtering, reads used by miRDeep2 quantification process; c: reads with at least one and at most five reported alignments, used by the miRDeep2 prediction process.

**Figure 1:**
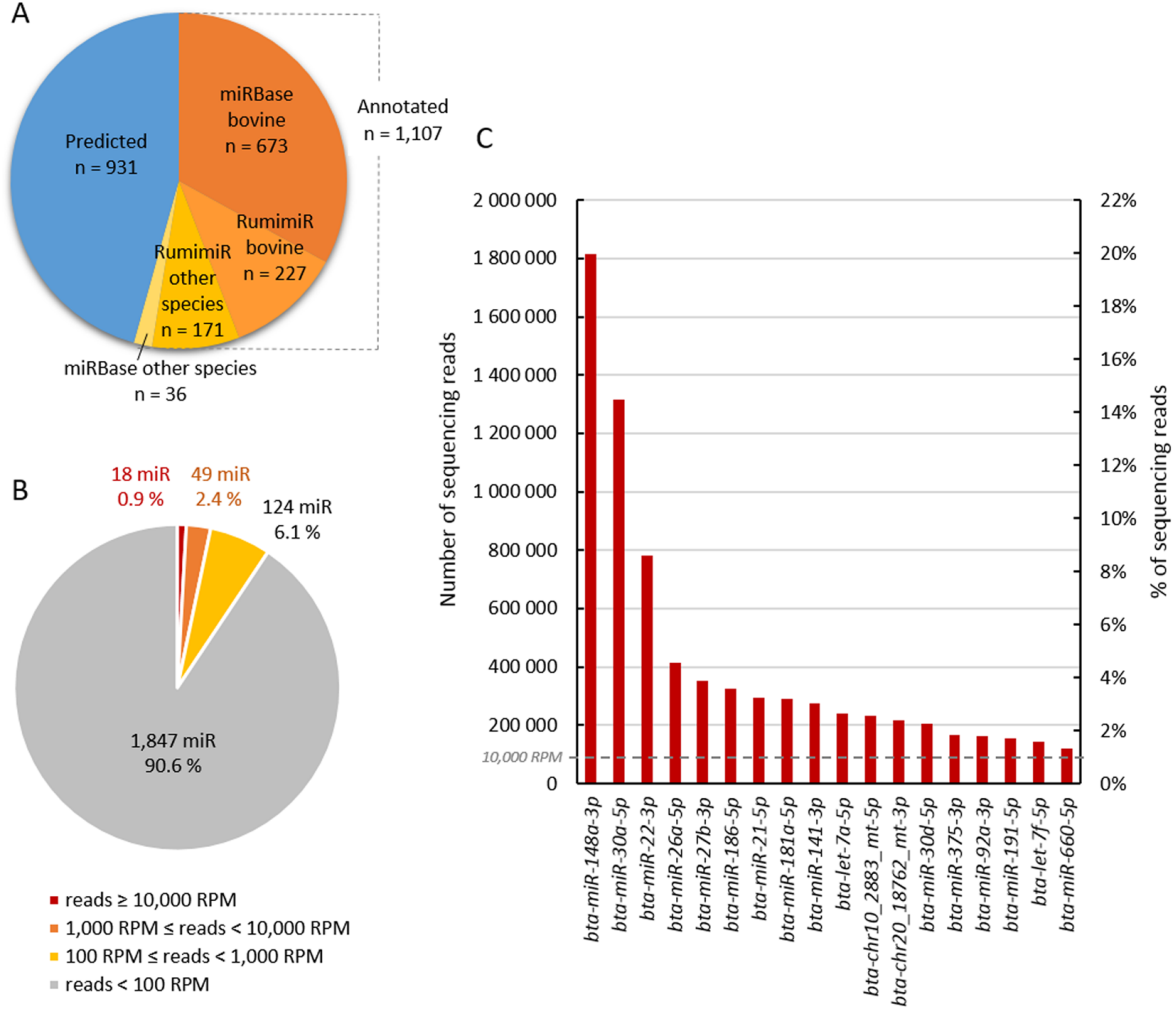
Description of the Holstein milk miRNome. Number of predicted and annotated microRNAs in Holstein milk, according to their annotation in the miRBase and RumimiR databases (**A**). Proportion of microRNAs according to their read abundance, on average, over the nine libraries (**B**) and major milk microRNAs, with abundancy equal to or greater than 10,000 RPM, which equals 91,879 reads (**C**). RPM: reads per million. n: number of microRNAs. %: relative number of microRNAs per range.

The analysis of these mapped sequences using miRDeep2 allowed the identification of 2,038 mature microRNAs in Holstein milk, corresponding to 1,107 annotated microRNAs and 931 predicted microRNAs (Fig. 1A; Supplementary Table). The annotated microRNAs corresponded to 673 bovine microRNAs referenced in both the miRBase^29^ and RumimiR^30^ databases, 227 bovine microRNAs referenced exclusively in the RumimiR database, and 171 and 36 microRNAs referenced in other species in RumimiR (caprine and ovine) and miRBase (12 species, especially Human and mouse).

Within the miRNome, microRNAs can be distributed according to their read abundance, expressed in reads per million (RPM). Approximately 90% of all microRNAs, corresponding to 1,847 microRNAs, are represented with a read abundance below 100 RPM (Fig. 1B). Two intermediate classes consisting of 124 and 49 microRNAs were present in milk with abundancies ranging between 100 and 1,000 RPM and between 1,000 and 10,000 RPM, respectively. The last group, comprising 18 microRNAs, exceeded 10,000 RPM (Fig. 1B,C). These 18 microRNAs corresponded to, in terms of number, 0.9% of microRNAs present in the miRNome and, in term of abundance, 82% of the miRNome. They could be considered the major milk microRNAs.

The three most abundant microRNAs *bta-miR-148a-3p*, *bta-miR-30a-5p* and *bta-miR-22–3p* (Fig. 1C) represent approximately 40% of the total reads. Among the major microRNAs, two are predicted (*bta-chr10_2883_mt-5p* and *bta-chr20_18762_mt-3p*) and mapped on the bovine chromosomes 10 and 20, respectively. They fulfil the prediction criteria of miRDeep2, passed through the Rfam filter, and the displayed miRDeep prediction scores equalled 5.5 and 1.5, respectively, and they did not match *Bos taurus* non-coding RNAs (lncRNA, rRNA, tRNA, snoRNA).

### The milk microRNA composition depends on the genetic background

To evaluate the impact of the genetic background on the milk miRNome, the high throughput microRNA profiles of two dairy cow breeds, Normande and Holstein cattle, were compared. A total of 33,810,306 raw reads were obtained, on average, per library of eight Normande cows, ranging from 3,065,890 to 71,359,415 raw reads, and then resulting in 915,487 to 19,656,622 mapped reads (Table 2).

**Figure 2:**
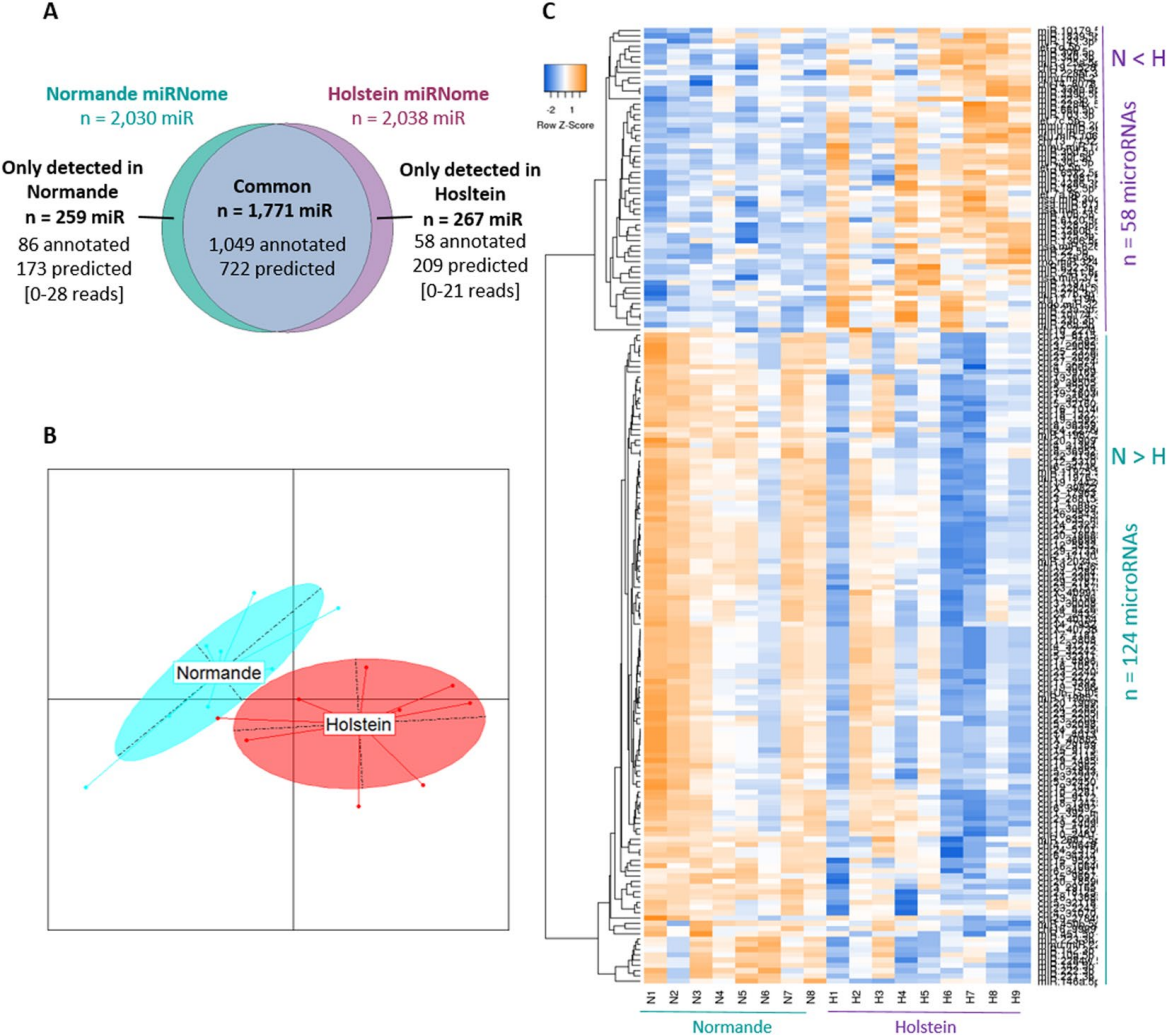
Comparison of Holstein and Normande milk miRNomes. Venn diagram depicting microRNAs present in Normande and Holstein milk (**A**), with the range of numbers of reads associated with microRNAs detected in only one of the two breeds in square brackets. Individuals were plotted according to their coordinates on the first two components of the principal component analysis (Normande in blue; Holstein in red). Inertia ellipses where 95% of individuals are likely to lie within characterize the dispersion of each breed around its centre of gravity (**B**). Heat map of pairwise Pearson correlation of the counts of milk microRNAs (p. adj. < 0.05) (**C**). miR: microRNA. n: number of microRNAs. In orange: higher level; in blue: lower level.

The Normande and Holstein milk miRNomes were compared. The total number of microRNAs identified in Normande cattle was 2,030, thus eight less than in Holsteins (Fig. 2A and Supplementary Table). The majority of the microRNAs were present in the milk of the two breeds, with 1,771 common microRNAs, comprised of 1,049 known and 722 predicted entities. However, these common microRNAs display variable abundancies between the two breeds. In particular, 14 microRNAs (13 annotated, 1 predicted) were ranked within a lower read abundance class (in RPM) in Normande milk than in Holstein, and 48 microRNAs (21 annotated, 27 predicted) were ranked within an upper RPM class in Normandes than in Holsteins (Supplementary Table).

Furthermore, some microRNAs were identified in the milk miRNomes of only one of the two breeds, with 267 and 259 microRNAs detected in Holstein or Normande milk only, respectively (Fig. 2A and Supplementary Table). All these microRNAs were poorly represented, with a number of reads up to 28, and were not present in all animals.

**Figure 3:**
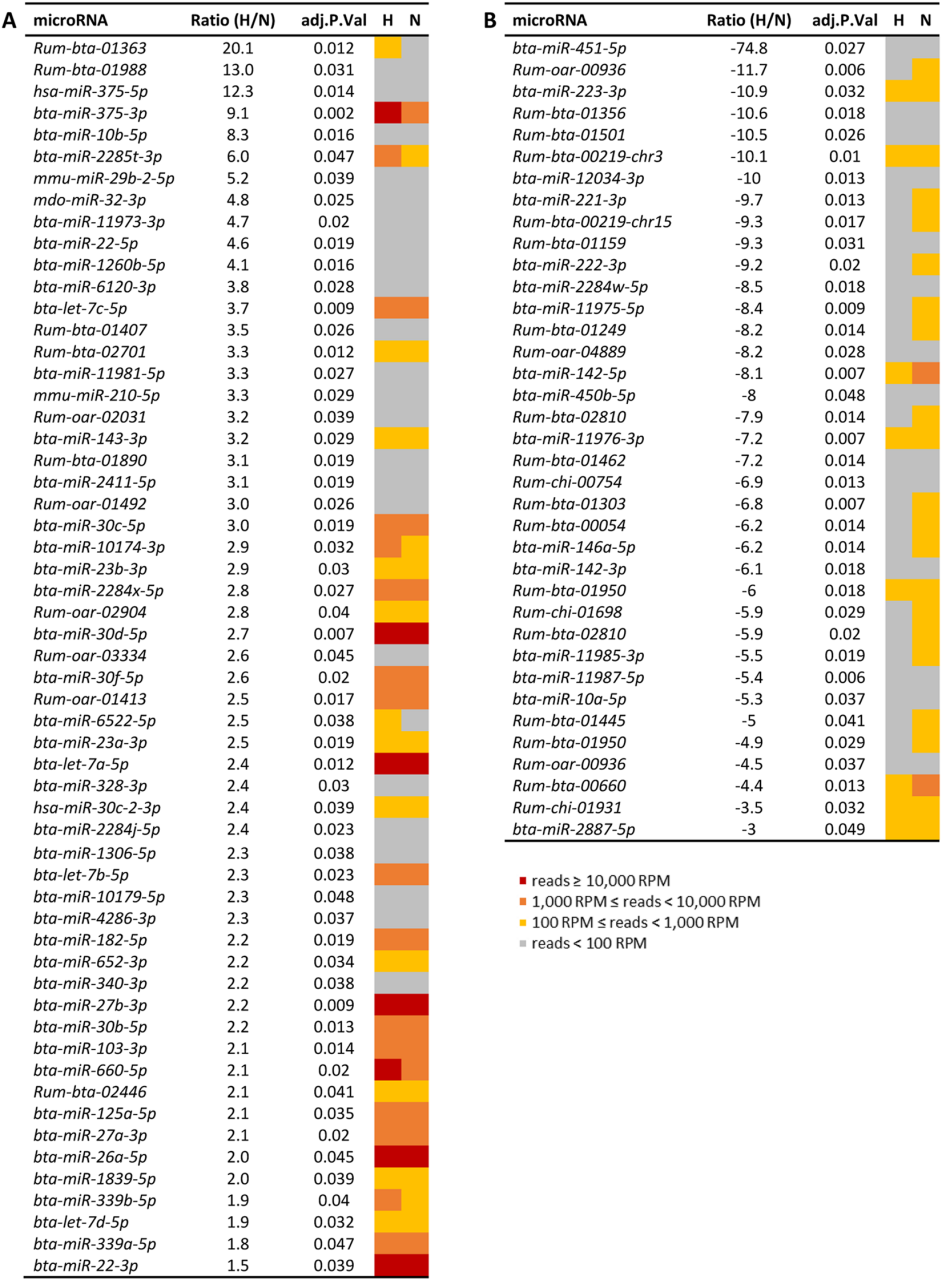
Annotated microRNA differentially present in milk according to breeds. Annotated microRNA with significant greater abundance in Holstein (**A**) or Normande (**B**) milk. H: Holstein, N: Normande; Ratio: abundance ratio; adj.P.Val: adjusted P-value. Classes of microRNAs according to their read abundance: red ≥ 10,000 RPM; 10,000 RPM > orange ≥ 1,000 RPM; 1,000 RPM > yellow ≥ 100 RPM; grey < 100 RPM.

To perform a comparative analysis of the microRNA read counts, the HTSFilter^31^ package was used to identify the threshold that maximizes the filtering similarity among samples. The identified threshold was equal to 168. The microRNAs whose expression is below this threshold are considered to generate a noisy signal and are then discarded from the analysis. Therefore, differential analysis was applied to 502 common microRNAs, whose maximal count across all individuals was higher than 168 reads. The statistical analysis revealed significant differences (p < 0.05) between milk miRNomes (Fig. 2B): 58 and 124 microRNAs are less and more abundant, respectively, in the milk of Normande than Holstein cows (Fig. 2C). Within the microRNAs less abundant in the Normande milk, 57 are annotated and display abundance ratios between 1.5 and 20.1 (Fig. 3A). Thirty-four of these 57 microRNAs are present with a read abundance above 100 RPM in Holstein milk. In particular, seven major microRNAs in Holstein milk (with RPM higher than 10,000 RPM) are involved: *bta-miR-22-3p*, *bta-miR-26a-5p*, *bta-miR-27b-3p*, *bta-miR-30d-5p*, *bta-miR-375-3p*, *bta-miR-660-5p* and *bta-let-7a-5p*, displaying abundance ratios from 9.1 to 1.5. Two microRNA families are particularly represented: the *let-*7 family with four members and the *miR-30* family with five members.

Among the 124 microRNAs more abundant in Normande milk, 37 are annotated, with variations abundance ratios between −3.0 and −74.8 (Fig. 3B). Only eight microRNAs are present with abundance higher than 100 RPM in Holstein milk: *bta-miR-223-3p*, *bta-miR-142-5p*, *bta-miR-2887-5p*, *bta-miR-11976-3p*, *Rum-bta-00219-chr3, Rum-bta-00660*, *Rum-bta-01950* and *Rum-chi-01931*.

### The milk microRNA profile varies according to age

**Figure 4:**
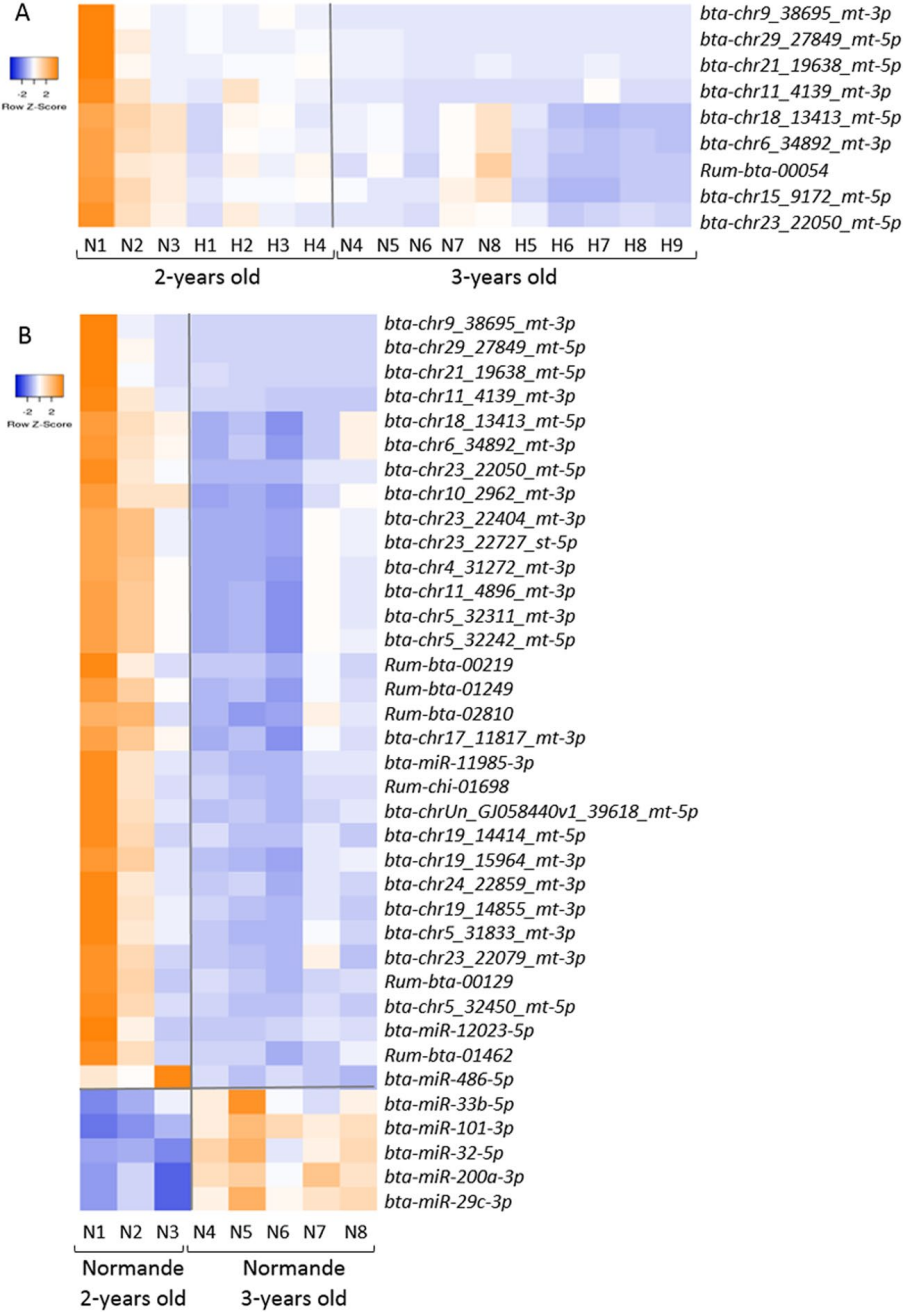
Variation of the milk miRNome according to age. Heat map of significantly different normalized read counts of milk microRNAs (p. adj. < 0.05) between 2-year-old and 3-year-old animals, among both breeds (**A**) or within the Normande breed (**B**). H: Holstein; N: Normande. In orange: higher level; in blue: lower level.

The Holstein and Normande cows were distributed into two age groups. The analysis of the milk miRNomes was extended to a study of the impact of the age. The differential analysis between groups of age of the two breeds highlighted nine microRNAs (one annotated and eight predicted microRNAs) with significantly higher abundance (p < 0.05) at 2 years of age compared to 3 years of age (Fig. 4A).

Intra-breed analyses were also performed showing no significant difference between age groups within the Holstein breed. In contrast, 37 microRNAs were present in significantly different levels (p < 0.05) according to age group within the Normande breed (Fig. 4B). In fact, five microRNAs are more abundant in the milk of 3-year-old rather than in 2-year-old Normande cattle, and 32 microRNAs (nine annotated, 23 predicted) are present at lower levels in 3-year-olds when compared to 2-year-old cows, seven of which also emerged from the age analysis that included both breeds.

### The milk miRNome is a partial reflection of the lactating mammary gland miRNome

**Figure 5:**
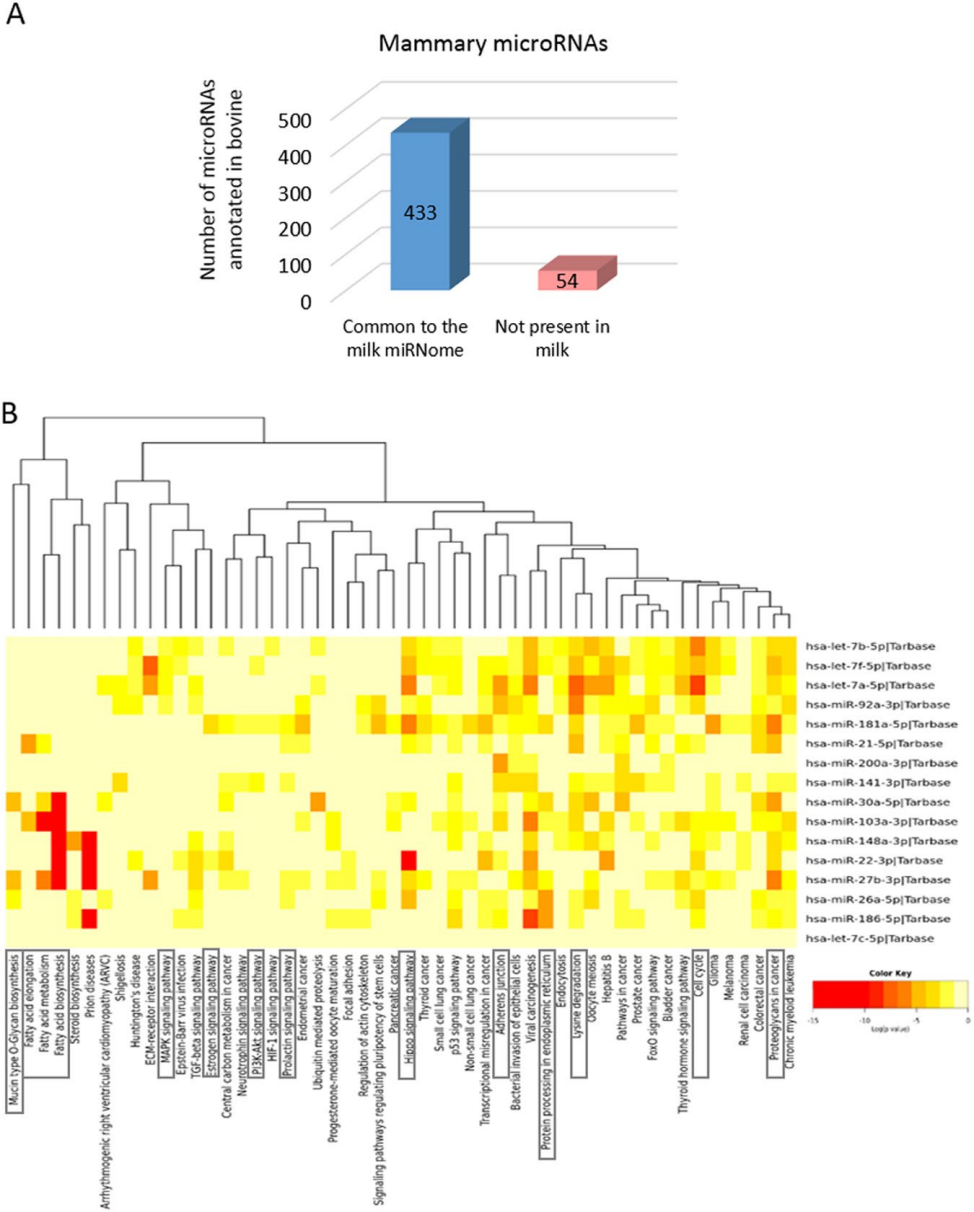
Comparison of Holstein milk and mammary gland miRNomes. Venn diagram depicting annotated microRNAs present in lactating mammary glands and milk (**A**). Heat map of the 16 most abundant microRNAs in both milk and lactating mammary glands versus significantly enriched functional union pathways, from Tarbase v7.0 and Diana mirPath v3.0 (**B**). miR: microRNA. According to the colour key, red indicates lower p values. In boxes: key pathways for mammary differentiation and lactation.

This Holstein whole milk miRNome was carried out in exactly the same way (RNA isolation, library construction and sequencing, analysis processes) as our previous study of the Holstein lactating mammary gland^32^, consisting of 654 annotated (487 microRNAs annotated in bovine, 167 in other species) and 679 predicted microRNAs. Amongst the 487 miRNA annotated in bovine, 433 (88.9%) were detected in milk (Fig. 5A).

Notably, 16 common microRNAs are present in the top 30 of both the milk and the mammary gland (Table 3), which include the 3 microRNAs most present in milk, *miR-148a-3p*, *miR-30a-5p* and *miR-22a-3p*. This miRNome comparison has also allowed the highlighting of microRNAs with contrasting levels of abundance between milk and the mammary gland. In fact, five microRNAs are particularly present in milk, whereas their expression is low in mammary tissue: *miR-30d-5p*, *miR-375-3p*, *miR-191-5p*, *miR-660-5p* and *miR-423-5p* (Table 3). In contrast, ten other microRNAs are strongly expressed in the mammary gland while their abundance in milk is low, in particular *miR-16-5p, miR-23a-3p, miR-126-5p, miR-143-3p* and *miR-200c-3p* (Table 3). Furthermore, an analysis of the common top 30 microRNAs, using Tarbase and Diana mirPath, has underscored the regulation of some key pathways for lactation. First, regulatory networks controlling milk fat synthesis and metabolism, and second, pathways related to protein processing in the endoplasmic reticulum, glycan biosynthesis and MAPK signalling (Fig. 5B).

**Table 3:** Comparison of the top 30 microRNAs of Holstein milk and lactating mammary glands.

	microRNA	Milk		Mammary gland	
		Rank	Reads	Rank	Reads
Most abundant microRNAs in both milk and mammary gland	*bta-miR-148a-3p*	1	211,987	1	73,408
	*bta-miR-30a-5p*	2	148,868	24	14,132
	*bta-miR-22-3p*	3	79,454	12	22,402
	*bta-miR-26a-5p*	4	48,350	2	69,491
	*bta-miR-27b-3p*	5	38,236	20	18,194
	*bta-miR-186-5p*	6	36,384	33	7,350
	*bta-miR-21-5p*	7	31,081	5	51,854
	*bta-miR-181a-5p*	8	30,827	36	6,554
	*bta-miR-141-3p*	9	26,984	18	19,658
	*bta-let-7a-5p*	10	25,862	3	66,625
	*bta-miR-92a-3p*	14	16,553	30	7,871
	*bta-let-7f-5p*	15	14,616	16	20,529
	*bta-let-7b-5p*	20	6,459	6	38,006
	*bta-miR-103-3p*	22	5,538	27	10,838
	*bta-miR-200a-3p*	23	5,051	25	13,369
	*bta-let-7c-5p*	30	2,573	28	9,822
microRNAs more abundant in milk than in the mammary gland	*bta-miR-30d-5p*	11	22,893	48	4,013
	*bta-miR-191-5p*	12	18,699	45	4,788
	*bta-miR-375-3p*	13	17,306	59	1,984
	*bta-miR-660-5p*	16	11,945	101	682
	*bta-miR-423-5p*	17	9,514	71	1,555
microRNAs more abundant in the mammary gland than in milk	*bta-miR-200c-3p*	34	2,045	9	27,254
	*bta-let-7g-5p*	40	1,646	15	20,894
	*bta-miR-29a-3p*	47	1,422	23	14,332
	*bta-let-7i-5p*	49	1,252	26	11,049
	*bta-miR-16-5p*	63	659	10	25,906
	*bta-miR-23a-3p*	66	579	8	30,292
	*bta-miR-143-3p*	68	573	4	60,696
	*bta-miR-200b-3p*	83	252	19	19,187
	*bta-miR-24-3p*	88	203	14	21,134
	*bta-miR-126-5p*	225	8	11	24,272

Ranks and average normalized reads of the microRNAs annotated in bovine divided into three groups: most abundant in both milk and the mammary gland, more abundant in milk than in the mammary gland and more abundant in the mammary gland than in milk.

## Discussion

The in-depth knowledge of bovine milk composition is a matter of ongoing interest, as well as the understanding of its variation according to genetic factors. In this context, we performed for the first time an exhaustive characterization of the miRNomes in the milk of Holstein and Normande cows, two dairy breeds with distinct genetic backgrounds and milk production features, in the purpose of highlighting differences in microRNAs between breeds associated with the regulation of milk synthesis.

This study provides an extensive description of the whole milk miRNomes, consisting of over two thousand microRNAs, of which half are annotated in ruminant species. Here, the deep sequencing data analysis allowed the identification of 207 new bovine microRNAs that had already been identified in other species. These findings resulted from the broad use of the miRDeep2 tool for all species and not only the bovines, which was complemented by the use of the latest update of miRBase database (v22) and the newly available RumimiR database. Furthermore, it allowed the prediction of 931 microRNAs, adding new elements to published data obtained by the analyses of the only annotated bovine microRNAs^22,23,33^.

The comparative analysis of the milk miRNomes between Holstein and Normande cattle provides an extended list of 1,771 known and predicted microRNAs shared between breeds, which corresponds to almost 87% of each miRNome. The two breeds have the same top 10 milk microRNAs. Five of them are also in the top 10 of the Fleckvieh^33^ and Brown Swiss^33,34^ breeds whole milk miRNomes: *miR-21-5p, miR-26a-5p*, *miR-30a-5p*, *miR-148a-3p* and *let-7a-5p*. This finding reinforces the likely importance of these microRNAs for lactation, particularly for *miR-148a-3p*, which has been consistently reported as the most abundant microRNA in the different milk fractions (fat, whey, and extracellular vesicles)^21^.

Three microRNAs, *miR-99a-5p, miR-200a-3p* and *miR-200c-3p*, present in the top 10 of the Fleckvieh^33^ and Brown Swiss^33,34^ breeds are not among the major microRNAs, albeit they are present at more than 1,000 RPM, in Holsteins and Normandes. The discrepancies between the top 10 may be due to differences between the breeds, and it would be interesting to explore them further. Nonetheless, the independent studies comparison remains sensitive, partly due to possible disparities between the RNA sequencing analysis and the microRNA identification processes. To be ensured of an effective breed impact and to overcome any technical bias, the systematic use of a reference milk miRNome in all research studies would be insightful. With the Holstein being the first world’s dairy breed, its routine addition in every high-throughput analysis on bovine would be relevant.

Furthermore, this study allows the introduction of new insights about the differences in the miRNA patterns between Holstein and Normande cattle, which was unknown until now, and could be related to differences in milk production and breed genetic backgrounds. Two sets of microRNAs, dependent on breed, are highlighted. One set consists of 267 and 259 microRNAs only detected in Holstein or in Normande milk, respectively, with very low abundance and not found in all animals. Their low presence reduces the impact of their potential biological role, and their absence in the milk of some individuals prevent their use as breed milk biomarkers. However, they could be used as relevant candidates for further investigations of tissues or fluids other than milk in the respective breeds to highlight breed-specific biomarkers as long as they display valuable expression levels.

More importantly, the other set of microRNAs is formed by 182 microRNAs present in both breeds but with significant differences in abundance (p < 0.05). The different profiles according to breeds would result from variations on microRNA regulatory mechanisms, while knowing that milk microRNAs are primarily synthesized in the mammary tissue^27^ and that their level in milk is linked to their expression in the mammary gland^28^. Thus, knowledge of the genes and pathways targeted by microRNAs displaying different abundance levels between breeds could be very useful for a better understanding of their roles in the regulation of milk production processes.

On the one hand, among the more abundant microRNAs in Holstein milk, the *let-7* and *miR-30* families are particularly represented. Interestingly, the *let-7* family is downregulated by the Wnt-β-catenin pathway^35^, as is *miR-375*^36^, which is far more abundant in Holstein than in Normande milk. The Wnt-β-catenin pathway is involved in mammary gland development, notably in alveolar morphogenesis during pregnancy^37^, and has a role in the terminal differentiation of the mammary epithelial cell, particularly in the maintenance of epithelial integrity, which is essential for lactation^38^. Furthermore, in mammary cells, oestrogen could induce the expression of the members of the *let-7* and *miR-30* families and regulate them by altering their rate of processing^39^. Tong *et al*. demonstrated that oestrogen has an effect on the proliferation of bovine mammary epithelial cells and on the modulation of the key components of the Wnt signalling pathway^40^. The regulation and interactions between important hormone-activated pathways for mammary gland function could differ between Holstein and Normande cattle, in association with differences in their milk production, and could thus affect expression of the different microRNAs highlighted in this study. The high expression of *let-7*, *miR-375* and *miR-30* in Holstein milk could be the result of specific regulation of the mammary signalling pathways in order to promote high milk production.

The *miR-221/222* cluster and the *miR-142* family are more prevalent in Normande milk. The *miR-221/222* cluster is involved in lipid metabolism in mammary epithelial cells^41^ and the regulation of lactose^42^. Chu *et al*. showed that inhibition of *miR-221* expression increased lipid content in mammary epithelial cells through elevation of the lipid synthesis enzyme FAS, while microRNA overexpression reduced lipid content^41^. The expression of *miR-221* and *miR-222* are correlated with genes strongly expressed in adipocytes and related to lipid metabolism^43^. The role of the *miR-221/222* cluster in lipid regulatory processes is obviously complex and needs further investigation, for example, regarding its specific deregulation in bovine mammary cells and to determine its impact on gene expression related to lipid pathways. Furthermore, *miR-142* may be involved in the regulation of the number of somatic cells in milk^42^. *miR-223*, also significantly more abundant in Normande than in Holstein milk, has been shown to interact with *miR-142* by upregulating its expression through transcription factors^44^ and could play a role in the mammary response to pathogens after parturition^45^. Milk yield is unfavourably genetically correlated with mastitis, notably in the Holstein breed, and largely correlated with the somatic cell score^46,47^. Differential regulation of microRNAs such as *miR-142* and *miR-223* according to cattle genetic background could influence this relationship, making these microRNAs possible candidates in strategies for the improvement of mastitis resistance.

Therefore, the *in silico* functional analysis performed for the targeted genes of the microRNAs with different levels in milk of Normande and Holstein revealed pathways related to the regulation of milk fat synthesis and metabolism, and protein processing in the endoplasmic reticulum. By being involved in the regulation of lipid and protein metabolism, these microRNAs could affect major lactation processes, and thus, their variation could lead to differences in milk production.

Milk is a daily ready access fluid, allowing the development of noninvasive identification and the analysis of biomarkers for production efficiency, health, physiological status, environmental impact and animal welfare state in dairy cattle^48^. Among milk biomarkers, microRNAs are relevant candidates with good stability and resistance that are arousing growing interest^21^. For example, seven microRNAs, including *miR-148a*, have been proposed to serve as quality control markers of milk products^16^. Here, this study also highlights microRNAs that could be used as breed indicators to help indicate fraud in labelling during cheese manufacturing. Our results permit a proposal of the dosage of a combination of three microRNAs, as *miR-375-3p*, *miR-660-5p* and *miR-142-5p*, to detect the presence of Holstein milk.

Moreover, the variable repartition of microRNAs among milk fractions (whey, fat, cells, vesicles) may result from distinct microRNA maturation or address processes that previously occurred in bovine mammary gland^9,21,49^. Their specific abundance level in some fractions might affect milk processing characteristics and also may have implications for neonatal or consumer health. Thus, the accuracy of the distribution of the significantly different microRNAs, such as *miR-375-3p* and *miR-142-5p*, among milk fractions between Holstein and Normande cows will help to refine their potential impact.

Furthermore, analysis of the milk miRNomes revealed nine microRNAs with significant differences in abundance level according to age. Then the intra-breed analysis revealed an age effect within Normande cattle, but not within Holsteins. However the average milk production of the 2-year-old Normande cows differs from that of 3-year-old Normande cows. The differences observed according to the age could not be distinguish to the difference of milk production.

The comparison of Holstein milk and lactating mammary glands highlighted 16 microRNAs highly present in both miRNomes. Half of them (*miR-21-5p*, *miR-26a-5p*, *miR-30a-5p*, *miR-92a-3p, miR-148a-3p*, *miR-186-5p, miR-200a-3p* and *let-7a-5p*) were also identified in the comparative analysis of bovine milk compartment miRNomes (fat, whey and cells) reported by Li *et al*.^9^, driving particular interest in these eight microRNAs for their likely critical regulatory roles in bovine mammary gland development, function and milk synthesis. However, this comparison revealed (i) that around 11% of the bovine annotated mammary microRNAs are not present in milk and (ii) microRNAs with contrasting abundance levels between milk and the mammary tissue. These results raised the issue of an incomplete correlation between the expression of microRNAs in mammary tissue and their presence in milk. Milk microRNAs primarily originate from mammary epithelial cells^21,27^. One of the reasons of this difference is that these microRNAs may not be expressed in the luminal epithelial cells, but in myoepithelial cells or adipocytes, and are not secreted in the lumen. Indeed, among microRNAs displaying noticeable lower levels in milk than in the mammary gland, *miR-126-3p* and *miR-143-3p* are defined as mammary basal cell type-specific, and *miR-23a-3p* and *miR-29a-3p* are significantly overexpressed in myoepithelial basal cells compared to luminal cells^50^. Therefore, the milk miRNome is more accurately a reflection of the miRNome of the mammary luminal cells.

In all, this work demonstrates that milk microRNA composition depends on dairy cow breed, thereby leading to the identification of possible microRNA regulators of breed-specific or general lactation processes, providing new insights into the possible mechanisms of milk production. Among the differences in microRNAs between Holstein and Normande cows, displaying high levels in milk, *bta-let-7c-5p* and *bta-miR-375-3p* are particularly interesting. In fact, their abundance levels are significantly higher in the milk of Holsteins than of Normandes; however, their presence in milk does not reflect their expression in mammary glands. *bta-let-7c-5p* is in the top 30 of mammary gland microRNAs, whereas *bta-miR-375-3p* is much less expressed. The mechanisms leading to this special enrichment of *miR-375* in milk compared to that in mammary tissue are unknown, and deciphering them would provide interesting elements to gain a better understanding of the regulation of lactation. In particular, answering the question of whether this enrichment in milk is also the case in the Normande breed would provide additional information towards a possible specific role of this microRNA in the Holstein breed. Moreover, variations in milk according to breed are also observed in the cattle mammary gland for these two microRNAs. Both are significantly more expressed in the mammary gland of Limousin than that of the Holstein breed^24^.

Using TargetScan, a comparison of the lists of predicted target genes regulated by *bta-miR-375-3p* and *bta-let-7c-5p* highlighted 440 common targets, presumed to be more repressed by these two microRNAs in Holstein than in the Normande breed. The analysis of these common targets, using DAVID, resulted in the identification of more than 300 associated biological pathways. Among them, the best represented are those involved in transcription regulation and inhibition, signal transduction, cell proliferation activation and cellular differentiation, which constitute core functions related to an active cell metabolism necessary for lactation. These two microRNAs might therefore play an important role in the repression of key pathways involved in the production of a milk rich in fat and protein, which specifically characterizes the Normande milk.

## Conclusion

In conclusion, using new generation sequencing, we performed the first study of milk miRNomes in Holstein and Normande breeds. This study provides an extended list of annotated and predicted microRNAs, which, on analysis, have shown that the milk microRNA composition depends on genetic background. MicroRNAs displaying significant differences in abundance between Holstein and Normande milk are known to be involved in the regulation of lipid metabolism, mammary morphogenesis and differentiation. The results of this comparative analysis provide information for a better understanding of the roles of microRNAs for the regulation of milk production processes in general and the relation to breed specificities. Therefore, milk microRNAs could be used as breed indicators for milk product manufacturing, on the one hand.

## Materials and Methods

### Experimental animals and sample preparation

Experiments reported in this study comply with the Institut National de la Recherche Agronomique ethical guidelines, in strict accordance with the EU Directive guidelines and regulations (EU Directive 2010/63/EU). The experimental protocol was approved by the French Ministry of Higher Education, Research and Innovation (Authorization APAFIS#3066-201511301610897v2).

To minimize the effect of the environment, all cows were raised together on the same experimental farm, with the same diet and rearing conditions. All animals were housed in INRA facilities, at the INRA Le Pin experimental farm (Normandy, France). During the winter period (from 10 days before calving to April), cows were kept indoors and received a total mixed ration, distributed once daily.

Cows were milked twice a day. Milk samples were collected from 17 healthy lactating primiparous cows: nine Holstein cows (four 2-year-olds, five 3-year-olds) and eight Normande cows (three 2-year-olds, five 3-year-olds). Whole milk samples were collected from one-time morning milking, around lactation day-68 (from day-48 to day-79), simultaneously for each age group for both breeds. Milk yield was recorded over a five-week production in the milking parlour (Boumatic-2050®) at each milking throughout the experiment. Milk fat, protein, lactose, urea, acetone and BHB content were determined by mid-infrared spectrometry (MilkoScan FT600, Foss, Hillerød, Denmark), and somatic cell counts (SCC) with a Fossomatic cell counter (Foss, Hillerød, Denmark) at the Normandy Dairy Milk Analysis Laboratory (LILANO, Saint-Lô, France).

### Total RNA isolation

Total RNA were isolated from 500 µl of frozen bovine whole milk samples, without cells and fat fractionation, using the RNA NOW kit (Ozyme), with overnight precipitation to guarantee a maximum yield of small RNA. The concentration and integrity of the RNA were assessed by spectrophotometry (Nanodrop™, ND-1000) and by using the RNA 6000 Pico Kit on a Bioanalyzer 2100 (Agilent Technologies, CA, USA). The RNA samples were stored at −80 °C until needed for further processing.

### Small RNA library construction and sequencing

Small RNA libraries were prepared using the Illumina® TruSeq® Small RNA Library Prep Kit (Illumina) with RNA isolated from the milk of each cow, according to the manufacturer’s instructions, with PCR amplification up to 15 cycles, by the INRA @BRIDGe platform (Jouy-en-Josas, France).

Single-read sequencing of libraries was carried out on two lanes on an Illumina HiSeq 4000 sequencer by the GenomEast Platform (IGMBC, Illkirch, France). RNA sequencing data were subsequently deposited in the Gene Expression Omnibus (GEO): GSE134670.

### Computational analysis of sequencing data

After cleaning adapters and filtering for their size (17–28 nt) with Cutadapt^51^, the RNA-seq data were analysed using miRDeep2 software^52^ as described in Le Guillou *et al*.^32^, with the bovine reference genome bosTau8 and miRBase release 22 (all species)^29^. The quantification results generated by the quantifier.pl miRDeep2 module were filtered with a custom Perl script parse_miRDeep2_outputs.pl (https://forgemia.inra.fr/sylvain.marthey/paqmir/blob/master/paqmir_postprocess_quantifier/parse_miRDeep2_output.pl) to eliminate any redundancy between mature microRNAs by assigning them to the precursors in the following order of priority: (1) mature known in the bovine species, (2) mature known in another species, and (3) mature predicted unknown. Additionally, microRNAs of the last two categories were searched with the new RumimiR database (v. June 2019)^30^ in order to identify ruminant microRNAs already described in the literature but not in the last version of miRBase (v.22), and this information was included for the annotation of quantified microRNAs. Predicted microRNAs were blasted against non-coding RNA (lncRNA, rRNA, tRNA, snoRNA) databases using RNAcentral (v.11)^53^.

### Statistical analysis

Differences in dairy traits recorded between experimental animals were compared using nonparametric Mann and Whitney statistical analyses, with Microsoft Excel software. Tests results were considered to be statistically significant when p-values were smaller than 0.05.

Prior to the statistical analysis of sequencing data, the filtering method described by Rau *et al*.^31^ was used to remove microRNAs that appeared to generate an uninformative signal. This method aims to identify the threshold that maximizes the filtering similarity among biological replicates, or in other words that where most genes tend to have either normalized counts lower than or equal to the cut-off point in all samples (i.e. filtered genes) or higher than the cut-off point in at least one sample (i.e. non-filtered genes). Tests for differential expression were only applied to microRNAs whose maximal count across all samples was higher than its threshold. This method was implemented under the Bioconductor HTSFilter package, V1.24.00^31^. The threshold value was found to be equal to 168 reads.

A principal component analysis of these data was performed using R software v3.5.1 (R Development Core Team, 2018, http://www.R-project.org) with the ade4 package v1.7.13^54^, followed by a differential expression analysis between milk miRNomes of the two breeds or the two age-groups with the Limma package, v3.37.10^55^. After a data normalization procedure with the TMM method^56^, a voom transformation (mean-variance modelling at the observational level), which computes (log-) counts per million using the effective library size, was intended to process RNA-Seq data prior to linear modelling in Limma. Then, in Limma, the lmFit function was used to fit row-wise linear models and fold changes were estimated using an empirical Bayes shrinkage procedure. The p-values were adjusted for multiple testing using the Benjamini and Hochberg method^57^, and those with an adjusted p-value < 0.05 were considered to be significant.

The same statistical analysis processes were also performed within each breed in order to evaluate the impact of intra-breed age. The threshold values estimated with HTSFilter are equal to 119 and 236 reads in Holstein and Normande, respectively.

Cluster analysis according to pairwise Pearson correlations between the sequencing counts of the significantly different microRNAs were performed using Heatmapper^58^.

### Pathway analysis of microRNA target genes

The pathway analysis of genes targeted by several microRNAs was performed using DIANA miRPath v.3.0^59^. Targets are listed from DIANA-TarBase (v.7)^60^, a database devoted to the indexing of experimentally supported microRNA targets. KEGG analysis^61^ was performed using the pathway union option, FDR correction, enrichment analysis method using the Fisher’s exact test (hypergeometric distribution) with a p-value threshold equal to 0.05.

For dedicated analysis, targets of individual microRNAs were predicted using TargetScan (v.7.2)^62^ and their functional analysis was performed using DAVID Bioinformatics Tools (v.6.8)^63,64^.

## Supplementary information

Supplementary Table.
